# Novel Use of a Bronchial Blocker in a Challenging Case of Congenital Diaphragmatic Hernia—A Case Report

**DOI:** 10.3390/children8121163

**Published:** 2021-12-09

**Authors:** Deepika Sankaran, Shinjiro Hirose, Donald Morley Null, Niroop R. Ravula, Satyan Lakshminrusimha

**Affiliations:** 1 Division of Neonatology, Department of Pediatrics, University of California at Davis, Davis, CA 95817, USA; dnull@ucdavis.edu (D.M.N.); slakshmi@ucdavis.edu (S.L.); 2 Division of Pediatric Surgery, Department of Pediatrics, University of California at Davis, Davis, CA 95817, USA; shirose@ucdavis.edu; 3 Division of Pediatric Anesthesiology, Department of Anesthesiology and Pain Medicine, University of California at Davis, Davis, CA 95817, USA; nrravula@ucdavis.edu

**Keywords:** congenital diaphragmatic hernia, bronchial blocker, ventilation–perfusion mismatch, hypoxic respiratory failure, single-lung ventilation, one-lung ventilation

## Abstract

The diagnosis of congenital diaphragmatic hernia (CDH) is associated with significant morbidity and mortality. Survival of neonates with CDH has improved recently, although the clinical course is complicated by sequelae of hypoplastic pulmonary parenchyma and vasculature, pulmonary hypertension, ventilation/perfusion (V/Q) mismatch, reduced pulmonary function and poor somatic growth. In this case report, we describe an infant with an antenatal diagnosis of CDH with a poor prognosis who underwent initial surgery followed by a tracheostomy but had a worsening clinical course due to a large area of ventilated but poorly perfused lung based on a V/Q nuclear scintigraphy scan. The emphysematous left lung was causing mediastinal shift and compression of the right lung, further compromising gas exchange. The infant had clinical improvement following bronchial blockade of the under-perfused left lung. This paved the way for further management with resection of the under-perfused lung lobe and continued clinical improvement. We present the novel use of selective bronchial blockade in a challenging case of CDH to determine if surgical lung resection may benefit the infant. We also review the physiology of gas exchange during the use of a bronchial occluder and the relevant literature.

## 1. Introduction

Congenital diaphragmatic hernia (CDH) is caused by a defect in the diaphragm, allowing the entry of abdominal contents into the thoracic cavity, thus impeding pulmonary parenchymal and vascular development [1,2]. Longitudinal follow-up of CDH survivors is complicated by ventilation/perfusion (V/Q) mismatch along with reduced pulmonary function [3]. A bronchial blockade is a technique utilized in adults and children for single-lung ventilation (SLV) to improve exposure during thoracic surgeries, and it has been successfully used in infants and neonates as well [4]. We report a challenging case of congenital diaphragmatic hernia (CDH) with hypoxic respiratory failure and compromised lung perfusion in the left lung, in which bronchial blockade was successfully used to assess responsiveness to SLV and to aid in decision-making regarding potential lung resection.

## 2. Case Presentation

A 27-year-old woman was diagnosed to have a fetus with left-sided CDH in her routine antenatal ultrasound (at 20 weeks gestation). Based on antenatal fetal imaging, the liver was in its thorax, the left lung was not visible, the right lung measured 1.95 × 1.67 cm and the lung-to-head ratio (LHR) was 1.275 (observed/expected LHR 29–33% [5,6], qualitative lung index/QLI 0.499), and percent predicted lung volume (PPLV) on fetal MRI was 20.5, all of which indicated poor prognosis [7]. Additionally, the fetal echocardiogram was suggestive of hypoplastic left heart syndrome (HLHS). The prenatal screening included amniocentesis with 46 XX karyotype and normal alpha-fetoprotein levels. The pregnancy was also complicated by polyhydramnios.

An appropriate-for-gestational-age female infant was delivered by emergent cesarean section for fetal bradycardia after initial induction of labor at 39 weeks gestation. At delivery, she was apneic and floppy, and immediate cord clamping was performed. Her airway was intubated one min after birth, and a Replogle tube was placed to decompress her stomach. Her Apgar scores were 2, 5 and 8 at 1, 5 and 10 min, respectively. Her initial neonatal intensive care unit (NICU) course included gentle mechanical ventilation, followed by bedside surgical repair of CDH two weeks after birth. A postnatal echocardiogram confirmed small left-sided cardiac structures. She also had pulmonary hypertension (PHT) with supra-systemic pulmonary pressures that were managed with inhaled nitric oxide (iNO), milrinone infusion and sildenafil. She required a peripherally inserted central catheter (PICC) for parenteral nutrition and a gastrostomy tube placement to allow enteral feeding. Her respiratory support was gradually weaned to low flow nasal cannula at 0.5 L/min with 100% O_2_, received Palivizumab and was continued on oral sildenafil for mild residual PHT. Her microarray was normal. She was discharged home (located at a higher altitude) at 2.5 months of age, only to be readmitted two days later when she presented to the local emergency room with fussiness and emesis and developed respiratory failure, requiring endotracheal intubation. Her brain natriuretic peptide (BNP) was elevated at 4650 pg/mL and was diagnosed with pulmonary hypertensive crisis.

During her second hospitalization in the NICU, she was extubated to nasal continuous positive airway pressure (CPAP) within three days after management with high settings on high-frequency oscillatory ventilation and iNO for hypoxic respiratory failure and PHT. However, she did not tolerate further wean in respiratory support, owing to worsening PHT. She had multiple episodes of PHT crisis with hypoxia and hypercarbia. To assess the V/Q status in her lungs, a nuclear medicine scintigraphy scan was performed at four months of age that showed 10% perfusion to the left lung in comparison to 90% to the right lung. A chest CT scan confirmed hypoplastic left lung. Cardiac catheterization at five months demonstrated worsening PHT, and she was restarted on iNO. Flexible bronchoscopy at five months showed gross narrowing of left mainstem bronchus and lobar bronchi. Due to continued PHT crises and an inability to remain extubated, she underwent a tracheostomy at 5.5 months. She had a Broviac^TM^ (CR Bard, Salt Lake City, UT, USA) catheter placed for central venous access. She received several courses of dexamethasone, with minimal change in her respiratory status.

**Figure 1:**
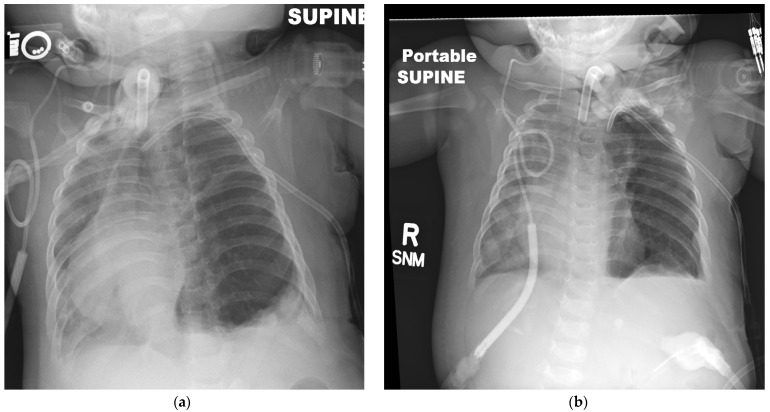
Radiographic findings in the infant. Chest X-ray appearance in the 6-month-old infant status-post repair of CDH, with a tracheostomy and a Broviac^TM^ catheter placement with significant emphysematous left lung with mediastinal shift (**a**). Conventional therapy with various ventilator modalities and dexamethasone did not result in any improvement (**b**).

At six months of age, the under-perfused left lung was emphysematous with a hyperinflated left lower lobe that caused a mediastinal shift to the right side (Figure 1a). This led to compression of the right lung, further compromising gas exchange. At this stage, due to the futility of all efforts to improve her hypoxic respiratory failure and the severe ventilation/perfusion mismatch (V/Q mismatch) in her left lung, a multidisciplinary discussion involving cardiology, pediatric surgery, pulmonology and neonatology was held to determine the next steps in her management. A trial on different ventilator modalities with higher positive end-expiratory pressure (PEEP) and another course of dexamethasone did not result in any improvement (Figure 1b). Given her labile nature with significant PHT and oxygenation concerns, there was concern if she would benefit from a lobectomy given the high risk of a procedure.

**Figure 2:**
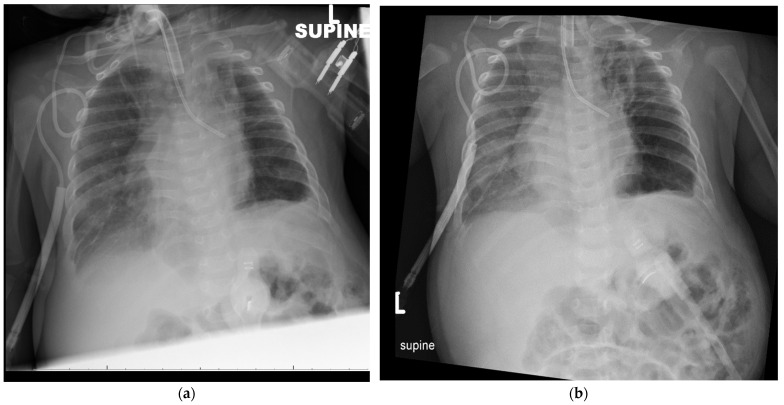
Chest radiographs after placement of bronchial blocker. Placement of a bronchial blocker in the left bronchus, resulting in better expansion of the right lung 6 h (**a**) and 24 h (**b**) after placement of blocker.

A decision was made to trial left main bronchial plugging by placing an inflatable 5Fr bronchial blocker to determine if improving V/Q mismatch by allowing better ventilation of the better perfused right lung segments may facilitate a decrease in respiratory support. We used two separate portable video laryngoscopes (VL), one equipped with a miller 1 blade and the other with a flexible fiberoptic bronchoscope (FFB). A 5 Fr Uniblocker (Fuji Systems, Tokyo, Japan) was guided through the glottic opening under visualization by the miller 1 blade VL. The bronchial blocker was guided into its final position and the balloon was inflated with the help of FFB. The position of the bronchial blocker was confirmed by a chest radiograph (Figure 2a,b). 

**Figure 3:**
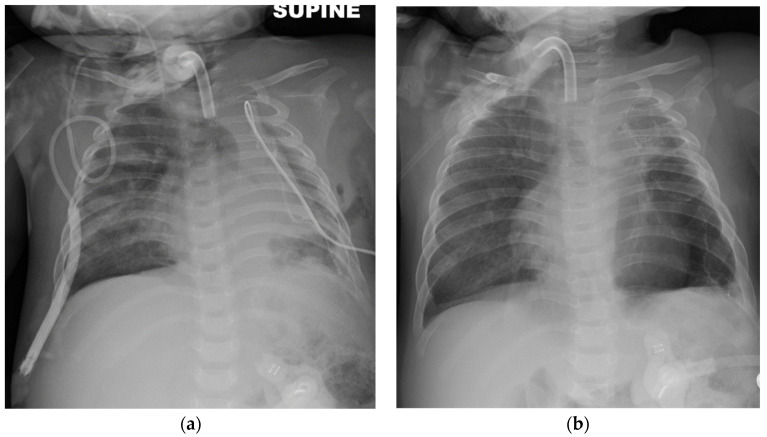
Improvement in right-lung expansion following surgery. (**a**) In the immediate postoperative chest X-ray and (**b**) chest X-ray close to discharge.

**Figure 4:**
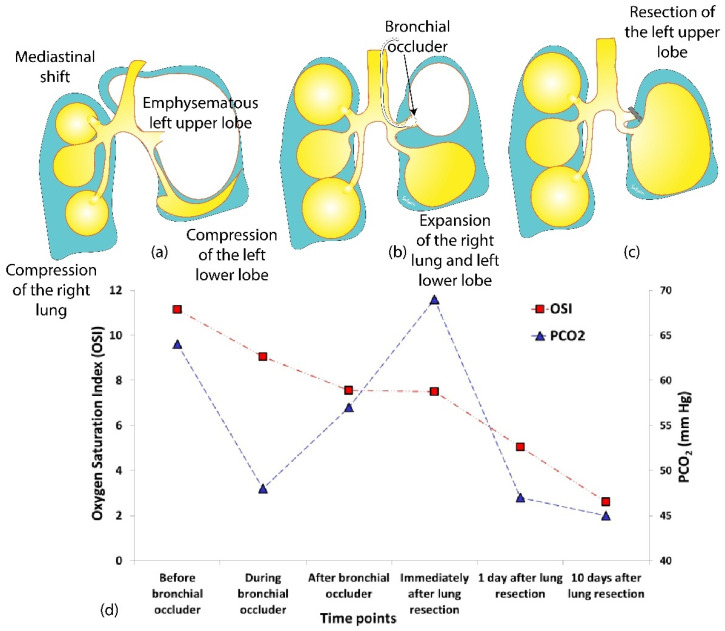
Physiological changes associated with bronchial occluder placement. (**a**) Prior to occluder placement, the emphysematous lobe compressed the left lower lobe and the right lung, resulting in high oxygen saturation index (OSI = mean airway pressure × FiO_2_ × 100 ÷ SpO_2_) and high PCO_2_. (**b**–**d**) Following bronchial occlusion and expansion of the right lung and the left lower lobe, FiO_2_ decreased from 0.47 to 0.37, resulting in improvement in OSI. (**d**) Following lung resection and recovery, FiO_2_ and OSI decreased further to 0.23 and 2.6, respectively.

The left bronchial blocker placement resulted in an immediate improvement of her respiratory status with a decrease in oxygen requirement and improved ventilation with weaning of ventilator settings, and better inflation of the right lung (Figure 2a,b). The adverse effects of the bronchial blocker placement included displacement, which required replacement under visualization by anesthesiology. Therefore, surgical exploration was subsequently performed by anterolateral thoracotomy, 80% of the emphysematous left upper lobe was resected (Figure 3), a small area of the left pulmonary sequestration was also identified and a chest tube was placed. Physiological and blood gas changes are shown in Figure 4. Respiratory support was weaned as tolerated, her chest X-ray improved (Figure 3b) and she was discharged home two months after surgery.

## 3. Discussion

CDH is characterized by hypoplasia of the lung parenchyma and vasculature [2]. These infants are at risk of severe pulmonary hypertension at birth and poor lung perfusion and function in the long run [8]. This is likely due to reduced ventilation in the ipsilateral side as the hernia and further compromised perfusion on the same side. Changes in lung ventilation and perfusion over time among CDH infants are unclear, with conflicting reports in the literature [9,10,11]. Arena et al. and Pal et al. demonstrated that improvement of the V/Q mismatch after CDH repair is predominantly due to a rapid increase in lung perfusion [9,12]. Dao et al. longitudinally evaluated V/Q mismatch in 212 CDH survivors and reported that V/Q in the cohort increased over time, which was driven by the progressive reduction in lung perfusion in the ipsilateral lung [3]. Furthermore, a higher V/Q ratio correlated with poor growth and poor functional status (New York Heart Association grade III or IV) [3]. The authors concluded that V/Q scans may be helpful in identifying CDH patients at risk of poor growth and functional status. In those infants with V/Q mismatch identified early on, it is not known if interventions to decrease ventilation to the under-perfused areas (such as selective bronchial blockade) may improve the respiratory status. Recently, electrical impedance tomography (EIT) has been evaluated as a useful tool to identify ventilation and perfusion defects in newborns [13,14]. EIT may have a potential role in the future for follow-up of infants with CDH.

The risk of subjecting an infant with CDH and PHT with a large emphysematous lobe to a lobectomy is high. The risks include complications of anesthesia (acute hemodynamic, neurotoxic, and ischemic changes) and acute derangements in hemostasis during surgery [15]. Post-pneumonectomy syndrome is an ominous complication caused by mediastinal shift following massive lung resection [16]. With progress in anesthesia and management approaches, the outcomes of lung resection have improved [15,17]. Due to the morbidity associated with anesthesia and surgery, we decided to test the physiological effects of lobectomy by occluding the bronchus. Our goal was not to perform a pneumonectomy; we were concerned that the left lung may require one, thus we occluded the left main bronchus rather than a lobar bronchus. To our knowledge, this is the first reported use of bronchial blockade in an infant with CDH with postoperative ipsilateral lung emphysema. Additionally, this is the first report of a prolonged bronchial blockade in an infant admitted in the NICU to assess respiratory improvement to aid decision making on the potential lung resection. In our patient, bronchial blockade allowed targeted ventilation and expansion of the better-perfused segments of the lung, which appeared to be primarily responsible for the clinical improvement observed with our patient (until the non-perfused segment of the lung could be surgically resected).

Single-lung ventilation (SLV) with the help of bronchial blockade has been utilized in adults and children during cardiothoracic surgery for lung isolation over the past three decades and has been safely used in infants and neonates [18,19]. With the advent of video-assisted thoracoscopic surgery, pediatric surgeons and anesthesiologists prefer SLV since it allows for better surgical exposure during procedures such as lung resections. By plugging the ipsilateral bronchus with a bronchial blocker (deflated ipsilateral lung), the contralateral lung is ventilated with SLV with lateral decubitus positioning of the patient. Some techniques for SLV include the use of double-lumen endotracheal tubes (ETT), endobronchial intubation with ETT, use of a bronchial blocker (e.g., Fogarty^R^ catheter, Edwards Lifesciences, Irvine, CA, USA), the collapse of the surgical lung by insufflation of carbon dioxide and retraction of the lung.

Bronchial blockade can be achieved by the placement of a Fogarty^R^ embolectomy catheter under direct visualization by rigid bronchoscopy, followed by inflation of the balloon to retain it in the desired location [19,20]. The blocker can be placed either intraluminally (via the ETT or tracheostomy tube) or extraluminally (separate from the ETT/tracheostomy tube) [20,21]. Bronchial diameters were measured in 250 children (from a two-day-old newborn to a 16-year-old adolescent) to enable appropriate sizing of the Fogarty^R^ catheters [22]. Alternatively, a balloon-tipped Arndt pediatric bronchial blocker (5Fr, Cook^®^ Critical Care, Bloomington, India) has also been used [23]. Reported complications (during intraoperative use) include atelectasis, which is usually transient, avulsion of the bronchial blocker cuff in the trachea, accidental fracture of its tip, displacement/dislodgement of the tip with compromised ventilation or even cardiac arrest and entrapment by a surgical stapler [18,20,24]. Entrapment of bronchial blockers was reported in a seven-month-old infant with congenital pulmonary airway malformation undergoing a thoracotomy and left lower lobectomy [25].

## 4. Conclusions

To conclude, the assessment of the. V/Q status in our CDH patient along with a multidisciplinary team approach towards managing the patient with complex physiology led to the trial of this novel technique in her management with a bronchial blockade, followed by a lung lobe resection. The physiological impact of a future lobectomy may be assessed by a bronchial occlusion. A bronchial blockade may be an effective way to assess the impact of future surgery on gas exchange and decompression of other regions of the lung. Infants with heterogeneous lung disease, such as post-operative CDH patients, may benefit from early and serial V/Q assessment to improve their pulmonary and overall outcomes.

## Data Availability

The data presented in this study are available in this article.
